# An extended KASP-SNP resource for molecular breeding in Chinese cabbage(*Brassica rapa* L. ssp. *pekinensis*)

**DOI:** 10.1371/journal.pone.0240042

**Published:** 2020-10-02

**Authors:** Shuangjuan Yang, Wentao Yu, Xiaochun Wei, Zhiyong Wang, Yanyan Zhao, Xiaobin Zhao, Baoming Tian, Yuxiang Yuan, Xiaowei Zhang

**Affiliations:** 1 Institute of Horticulture, Henan Academy of Agricultural Sciences, Zhengzhou, China; 2 College of Life Science, Zhengzhou University, Zhengzhou, China; Huazhong University of Science and Technology, CHINA

## Abstract

Kompetitive allele-specific PCR (KASP) is a cost-effective single-step SNP genotyping technology, With an objective to enhance the marker repertoire and develop high efficient KASP-SNP markers in Chinese cabbage, we re-sequenced four Chinese cabbage doubled haploid (DH) lines, Y177-47, Y635-10, Y510-1 and Y510-9, and generated a total of more than 38.5 billion clean base pairs. A total of 827,720 SNP loci were identified with an estimated density of 3,217 SNPs/Mb. Further, a total of 387,354 SNPs with at least 30 bp to the next most adjacent SNPs on either side were selected as resource for KASP markers. From this resource, 258 (96.27%) of 268 SNP loci were successfully transformed into KASP-SNP markers using a Roche LightCycler 480-II instrument. Among these markers, 221 (85.66%) were co-dominant markers, 220 (85.27%) were non-synonymous SNPs, and 257 (99.6%) were newly developed markers. In addition, 53 markers were applied for genotyping of 34 *Brassica rapa* accessions. Cluster analysis separated these 34 accessions into three clusters based on heading types. The millions of SNP loci, a large set of resource for KASP markers, as well as the newly developed KASP markers in this study may facilitate further genetic and molecular breeding studies in *Brassica rapa*.

## Introduction

Chinese cabbage (*Brassica rapa* L. ssp. *pekinensis*), which contains an AA genome, is one of the most important vegetable crops worldwide and is a valuable plant for genome evolution study in *Brassica* species [1, 2]. It was speculated Chinese cabbage was derived from a hybrid of Taisai and Turnip, and evolved from non-heading type, half opened head type to heading type [3, 4]. The heading type Chinese cabbage can further divided into overlapped, closed and spiral head types [5]. A phylogenetic tree based on resequencing data of 199 *B*.*rapa* accessions showed that the Chinese cabbage group was positioned at a more distant point from the root than other groups, such as turnip, sarson, pak choi, caixin and zicaita [6]. Other studies also confirmed that Chinese cabbage was distinct from other subspecies in genus *B*.*rapa* [2, 7].

Molecular markers are highly useful for genetic and genomic studies, such as linkage map construction, map-based cloning, marker-trait associated analysis, genetic diversity analysis and marker-assisted breeding for crop improvement [8–13]. Single nucleotide polymorphisms (SNPs) are the most abundant type of genetic variation in both animal and plant genomes, and have become the marker of choice in large-scale genotyping applications [14–16]. With the reduced cost of next-generation sequencing (NGS) technologies, an increasing number of SNP loci have been detected in many crops [17–19]. However, the majority of these SNP loci have neither been validated nor converted into robust markers. One of the major obstacles in utilizing SNP markers has been the efficiency and cost of genotyping compared to assessing other types of polymorphisms [20]. Recently, the Kompetitive allele-specific PCR (KASP) genotyping assay, developed by LGC Limited, has emerged as an attractive technology for SNP genotyping [21, 22].

KASP is a PCR-based novel homogeneous fluorescent SNP genotyping system. The major advantage of KASP is the improved cost-effectiveness because it is both less expensive and more reliable than other marker technologies, including other sequence-based markers, such as TaqMan [23]. In addition, KASP provides great flexibility in the number of SNPs and genotypes to be used for assays [7, 23, 24]. Since the advent of KASP, it has been well adopted and developed in wheat, rice, maize, chickpea, soybean, cucumber and many other crops [8, 9, 21–23, 25].

With an objective to enhance the marker repertoire and develop high efficient markers in Chinese cabbage, we re-sequenced four Chinese cabbage DH lines and conducted a genome-wide survey for SNPs. We established a KASP-SNP resource and converted 258 SNP variations into robust KASP markers. Furthermore, 53 of the 258 KASP markers were validated in 34 *Brassica rapa* lines. The genome-wide catalog of SNPs and the newly developed KASP markers will be valuable resources for the *Brassica* research community.

## Materials and methods

### Plant materials

Four Chinese cabbage doubled haploid (DH) lines, Y177-47, Y635-10, Y510-1 and Y510-9, were used for whole-genome re-sequencing with paired-end strategy on the Illumina platform. To validate the utility of these KASP markers, an additional panel of 29 accessions of *Brassica rapa* were used, including 3 Pak-choi (*Brassica rapa* L. ssp. *chinensis*), 2 flowering Chinese cabbage (*Brassica rapa* L. ssp. *chinensis* var. *parachinensis*), 2 Zicaitai (*Brassica rapa* L. ssp. *chinensis* var. *purpurea*), and 22 Chinese cabbage (S1 Table). All those accessions were DH lines or inbred lines, and were obtained from the Institute of Horticulture, Henan Academy of Agricultural Sciences, Zhengzhou, China.

### DNA extraction and re-sequencing

All plants were grown in farm field. Young leaves of 14-day-old seedlings were collected for DNA extraction. Genomic DNA of all accessions was extracted from young leaves using a modified CTAB method [26].

The sequencing libraries were constructed following the manufacturer’s instructions (Illumina Inc.). The Illumina HiSeq 2000 platform was used to generate 100-base paired-end reads for Y177-47 and Y635-10, while 150-base paired-end reads were generated for Y510-1 and Y510-9 on the Illumina HiSeq X Ten platform. The raw data were deposited in the Sequence Read Archive (SRA) in NCBI as SRP221414.

### SNP discovery and validation

After pre-processing of reads, where adapter sequences were removed and low quality reads were discarded, clean reads generated from the four accessions were mapped to the *B*. *rapa* reference genome V1.5 with Burrows-Wheeler Aligner (BWA 0.7.12-r1039) using default parameters [27]. The reference was downloaded at http://brassicadb.org/brad/datasets/pub/Genomes/Brassica_rapa, which consists of 10 chromosomes and 40357 scaffolds. The SAMtools software package (version 1.3.1) was used to call SNPs across all samples simultaneously [28]. Only SNPs that met all the following criteria were retained: a) minimum mapping quality > = 30; b) read depth of each genotype > = 5; c) the reference or variant allele was not an N; d) no more than one variant allele existed; and e) the genotype of any accession must be homozygous, the SNPs loci with any heterozygous genotype were filtered out. SNP annotation was performed with the ANNOVAR software [29].

To validate SNPs identified from whole genome re-sequencing, 142 of them were selected for PCR amplification and Sanger sequencing. Primers were designed using the Primer Premier 5.0 program [30] for at least one putative SNP. DNA samples of the four sequenced Chinese cabbage were used as templates for PCR amplification. PCR amplifications were carried out in a 20 μl reaction volume containing 2 μL (50 ng/μL) of DNA template, 0.1 μL Taq DNA polymerase (5 U/μL, Vazyme Biotech Co.Ltd, Beijing, China), 2 μL 10× PCR Buffer (Vazyme Biotech Co.Ltd, Beijing, China), 0.4 μL dNTPs (10 mmol/L, Vazyme Biotech Co.Ltd, Beijing, China), 1 μL (10 μmol/L) each of the forward and reverse primers, and 13.5 μL ddH_2_O. The PCR started with an initial denaturation at 94°C for 5 min, followed by 35 cycles of 94°C for 50 s, 56°C for 50 s, 72°C for 50 s, and a final extension at 72°C for 10 min. Sequencing results were analyzed by DNASTAR v7.1.0 (http://www.dnastar.com/).

### KASP primer design and validation

For each selected SNP, two allele-specific forward primers and one common reverse primer were designed using the Primer Premier 5.0 program [30] according to the standard KASP guidelines. The two allele-specific primers were added with the standard FAM (5'-GAAGGTGACCAAG-TTCATGCT-3') and HEX (5'-GAAGGTC-GGAGTCAACGGATT-3') tails respectively at the 5' end.

The newly developed KASP-SNP markers were validated by examining polymorphisms among the four sequenced Chinese cabbage lines. Before PCR application, the primer assay mixture was prepared, which comprised 46 μL ddH_2_O, 30 μL common reverse primer (μM), and 12 μL of each tailed allele-specific forward primer (μM). KASP assays were carried out in 96-well formats using Roche LightCycler 480-II instrument (Roche Applied Sciences, Beijing, China), and set up as 8 μl reaction volume (1.5 μL of DNA template with a concentration of 60 ng/μL, 4 μL of 2× KASP master mixture, 0.14 μL of primer assay mixture, and 2.36 μL ddH_2_O). PCR cycling was performed using the following protocol: hot start at 94°C for 15 min, followed by ten touchdown cycles (94°C for 20 s; touchdown at 61°C initially and decreasing by 0.6°C per cycle for 60 s), followed by 26 additional cycles of annealing (94°C for 20 s; 55°C for 60 s) (https://biosearch-cdn.azureedge.net/assetsv6/running-KASP-on-Roche-LC480.pdf). Fluorescence was read by the LightCycler480II and analyzed using the LightCycler 480 Software (Version 1.5.1).

### Overlap with KASP markers of previous studies

The 50 bp forward flanking sequences of RNA-based SNPs were downloaded from the article reported by Paritosh et al. [31]. We blasted these SNP flanking sequences against the *B*. *rapa* reference genome V1.5. Only the best-hit alignment (alignment length equals 50, sequence identity equals 100%) was retained. The SNP identifier was recorded using the format chr_position, for example, A02_1646719 indicates that there is a single nucleotide polymorphism in physical position 1,646,719 of chromosome A02. Similarly, the allele-specific forward primer sequence of KASP-SNP markers from the article reported by Su et al. [7] were also blasted against the reference genome to determine the SNP loci. These two sets of KASP-SNP markers were compared with those developed in this study.

### Diversity analysis

Twenty nine *Brassica rapa* accessions were genotyped using 53 randomly selected KASP-SNP markers. Genotypes of these 29 Chinese cabbage accessions together with 4 DH lines used for re-sequencing and the reference material Chiifu-401-42 were used for diversity analysis. For each marker, minor allele frequency (MAF), the total number of alleles, genetic diversity, heterozygosity and the polymorphism information content (PIC) were calculated with PowerMarker software (version 3.25) [32]. The CSChord (1967) genetic distancematrix of 34 *Brassica rapa* accessions based on 53 markers was calculated using PowerMarker software V3.25. The dendrogram was drawn by MEGA (version 5.1) using neighbor-joining algorithm (http://www.megasoftware.net/download_form).

## Results

### Sequence analysis and SNPs identification

Whole genome re-sequencing yielded approximately 62, 56, 99 and 94 million raw reads (Table 1), which represented approximately 12.80-, 11.59-, 30.63- and 29.24-fold genome coverage based on the estimated genome size of 485 Mb [33]. After quality control and filtering of the raw reads, clean reads of each sample were then mapped to the *B*. *rapa* reference genome (version 1.5) [33]. The mapping results showed that 93.37–95.31% of the clean reads could be mapped to the reference genome, and 67.28–81.43% were uniquely mapped reads (Table 1), indicating the high quality of the sequencing data.

**Table 1:** Summary of whole-genome re-sequencing data of four Chinese cabbage DH lines.

Catalog	Y177-47	Y635-10	Y510-1	Y510-9
**Raw reads**	62,060,798	56,204,878	99,046,946	94,539,100
**Raw bases**	6,206,079,800	5,620,487,800	14,857,041,900	14,180,865,000
**Coverage of *B*.*rapa* genome**	12.80 ×	11.59 ×	30.63 ×	29.24 ×
**Clean reads**	51,216,196	48,404,060	97,336,870	93,259,150
**Clean bases**	5,121,619,600	4,840,406,000	14,600,530,500	13,988,872,500
**Mapped reads**	47,820,291	45,246,339	92,333,755	88,885,296
**Mapped percentage (%)**	93.37	93.48	94.86	95.31
**Uniquely mapped reads**	34,456,390	32,677,844	79,264,088	73,501,968
**Mapped percentage (%)**	67.28	67.51	81.43	78.81

**Fig 1:**
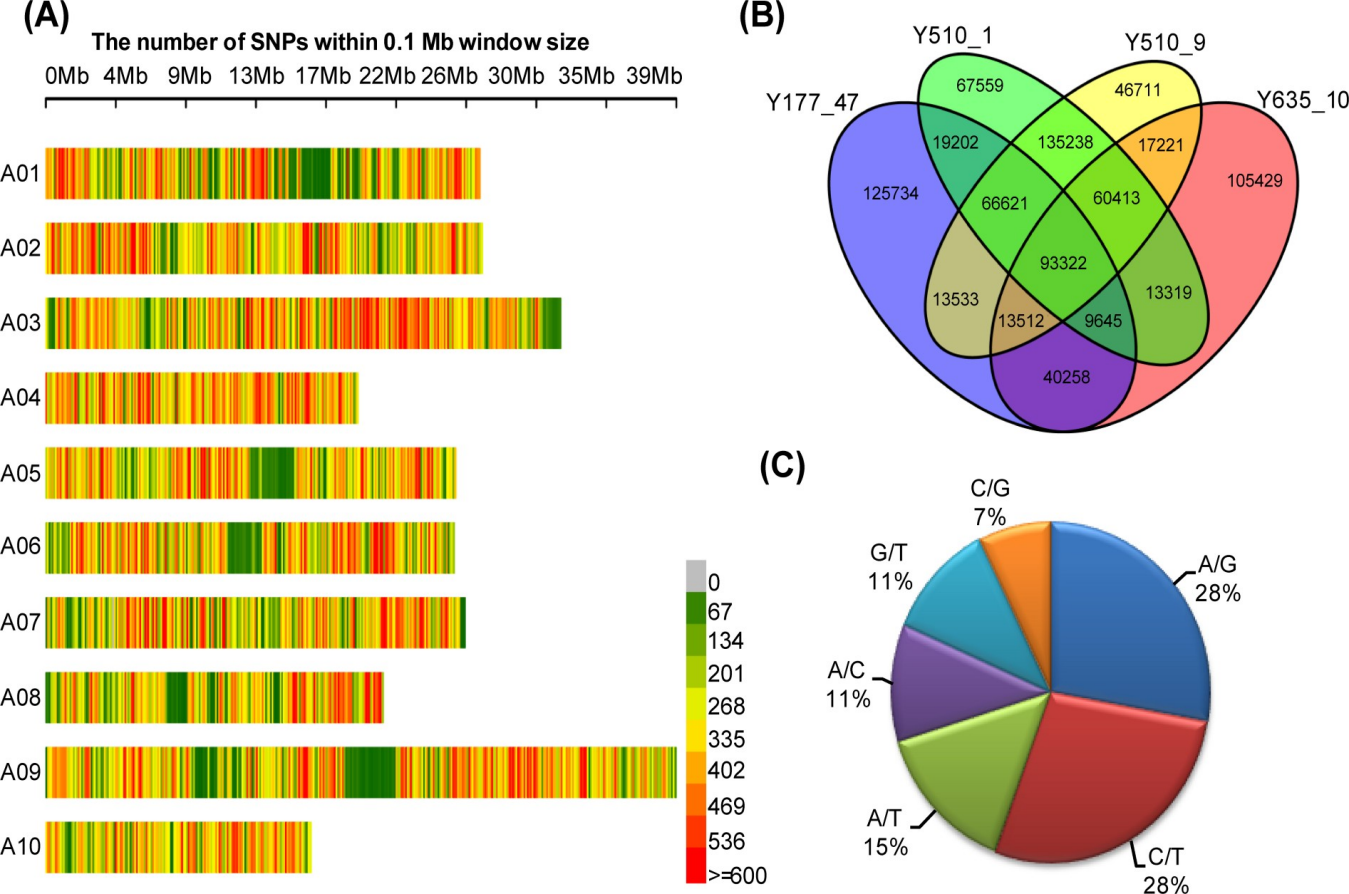
Number and type of SNPs identified in Chinese cabbage. (A) Distribution of SNPs in the pseudo-chromosomes of Chinese cabbage. (B) Venn diagram of SNPs among four Chinese cabbage DH lines. (C) Summary of SNP types identified in this study.

Through comparisons with the reference genome of the inbred Chiifu-401-42 (V1.5), we detected a total of 827,720 SNPs distributed among all ten chromosomes (Fig 1A), with an overall density of 3,217 SNPs/Mb (S2 Table). Among all these variations, 381,827, 353,119, 465,319 and 446,571 SNPs were detected in Y177-47, Y635-10, Y510-1 and Y510-9 compared to the reference genome, and 125,734, 105,429, 67,559 and 46,711 were specific to respective accessions (Fig 1B). Through pairwise comparison among the four DH lines and the reference material, Y177-47 and Y510-1 possessed the largest number of SNPs (469,566), while Y510-1 and Y510-9 showed the least number of SNPs (200,702) (S3 Table), which is accordance with the genetic distance between them. Of the 827,720 putative SNPs, the proportions of transitions were 28% A/G and 28% C/T (Fig 1C). Additionally, the proportions of transversions were 15% A/T, 11% A/C, 11% G/T and 7% C/G (Fig 1C. The percentage of transition variations (56%) was approximately 1.26 times the percentage of transversion variations (44%), close to the ratio in previous studies of *Brassica rapa* [2, 30].

To assess the validity of these *in silico* SNPs, a total of 41 primer pairs were designed for Sanger sequencing, and 30 primers yielded clear and single bands of the expected fragment size from the four original Chinese cabbage DH lines (S4 Table). These 30 primers targeted 142 putative SNP loci. Sanger sequencing results of all these 142 SNPs were completely consistent with the *in silico* genotype of each accession, demonstrating the high accuracy of the SNP dataset (S5 Table).

### Establishment of KASP-SNPs resource

In order to establish a KASP-SNPs resource, we filtered for SNP loci with at least 30 bp to the next most adjacent SNPs on either side. As a result, 387,354 SNPs were obtained, which could serve as resource for KASP-SNPs. All these SNPs and their flanking 30 bp sequence were supplied in S1 Appendix. According to their locations in the genome, all of these 387,354 SNPs were classified into several categories. As shown in Table 2, 63.79% (247,104) were identified in genic regions, while the other 36.21% (140,250) were in intergenic regions according to the *B*.*rapa* V1.5 genome annotation. A total of 190,962 (49.30%) SNPs were identified in expressed regions or splicing regions, and 56,142 (14.49%) were annotated in introns. In particular, 33,582 (8.67%) SNPs were classified as non-synonymous SNPs resulting in amino acid changes, and 443 (0.11%) SNPs gave rise to variants in stop codons or splicing regions, also altering the amino acid sequences of coding genes.

**Table 2:** Classification of KASP-SNPs resource in genic and intergenic regions.

Regions	Variants	No. of SNPs	Proportions(%)
**Genic region**	**non-synonymous**	33,582	8.67
	**synonymous**	59,875	15.46
	**stopgain**	230	0.06
	**stoploss**	65	0.02
	**splicing**	148	0.04
	**upstream**	47,873	12.36
	**downstream**	40,386	10.43
	**upstream/downstream**	8,803	2.27
	**Intron**	56,142	14.49
**Intergenic region**		140,250	36.21

### Development and validation of new KASP-SNP markers

To develop novel KASP-SNP markers, we selected 268 SNP loci to design markers from the above resource based on two criteria. First, SNPs are evenly distributed across the whole genome with a density of 1 marker/Mb. Second, the SNPs located in the exonic region with non-synonymous variation were preferred.

**Fig 2:**
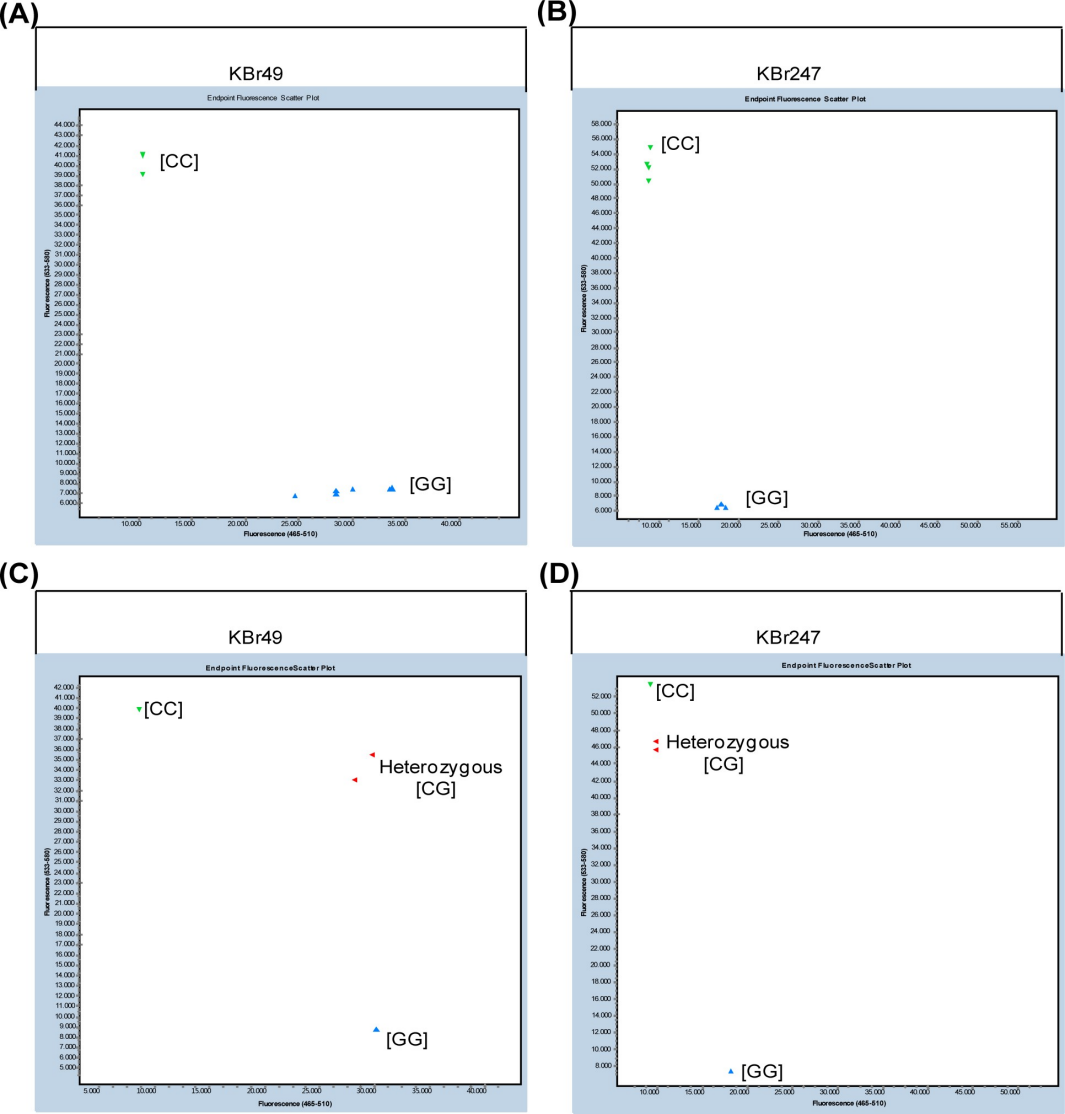
KASP graphs of two different polymorphic SNP types. Co-dominant (A) and dominant (B) KASP marker screened against the four sequenced Chinese cabbage lines (each with two replicates). Co-dominant (C) and dominant (D) KASP marker validated with two homozygous and two heterozygous genotypes.

In total, 258 (96.27%) worked well and gave rise to polymorphisms, which categorized the four sequenced genotypes into two clusters, while the other ten primers produced no signal. All the validated KASP markers were named using the format KBr1 (for KASP marker of *Brassica rapa* No.1), and the primer sequences and other information are listed in S6 Table. Among the 258 polymorphic markers, 221 (85.66%) were classified as co-dominant markers (Table 3), which produced both strong signals for each of the SNP genotypes. The other 37 (14.34%) were classified as dominant markers, which means that one of the SNP genotypes produced a strong signal, while the signal of the other SNP genotype was considerably weaker (Table 3). For example, the KASP marker KBr49, which represented SNP located in the A02 chromosome (26,929,619 G/C), produced an equal strength of the fluorescence signal for both the homozygous genotype [CC] and [GG] (Fig 2A), and the heterozygous genotype [CG] clustered almost along the diagonal line, which was clearly distinguished from the two homozygous genotypes (Fig 2C). Thus, KASP markers like KBr49 were deemed co-dominant markers. For the dominant marker KBr247 (A10_8107014 G/C), the signal of the homozygous genotype [GG] was considerably weaker than that of the genotype [CC] (Fig 2B), and the cluster of the heterozygous genotype [CG] was adjacent to the stronger homozygous genotype [CC] (Fig 2D), which means that this type of marker could differentiate the two homozygous genotypes but could not distinguish the heterozygous genotype from one of the homozygous genotypes. Thus, this type of KASP marker were designated the dominant marker.

**Table 3:** Distribution and number of KASP-SNP markers in Chinese cabbage.

Chr	Codominant	Dominant	Total SNPs	Length(Mb)	SNPs/Mb
**A01**	22	1	23	26.79	0.86
**A02**	22	4	26	26.94	0.97
**A03**	24	2	26	31.77	0.82
**A04**	18	4	22	19.27	1.14
**A05**	22	2	24	25.30	0.95
**A06**	18	4	22	25.21	0.87
**A07**	25	3	28	25.88	1.08
**A08**	22	4	26	20.83	1.25
**A09**	30	11	41	38.88	1.05
**A10**	18	2	20	16.41	1.22
**Total**	221	37	258	257.27	1.00

**Fig 3:**
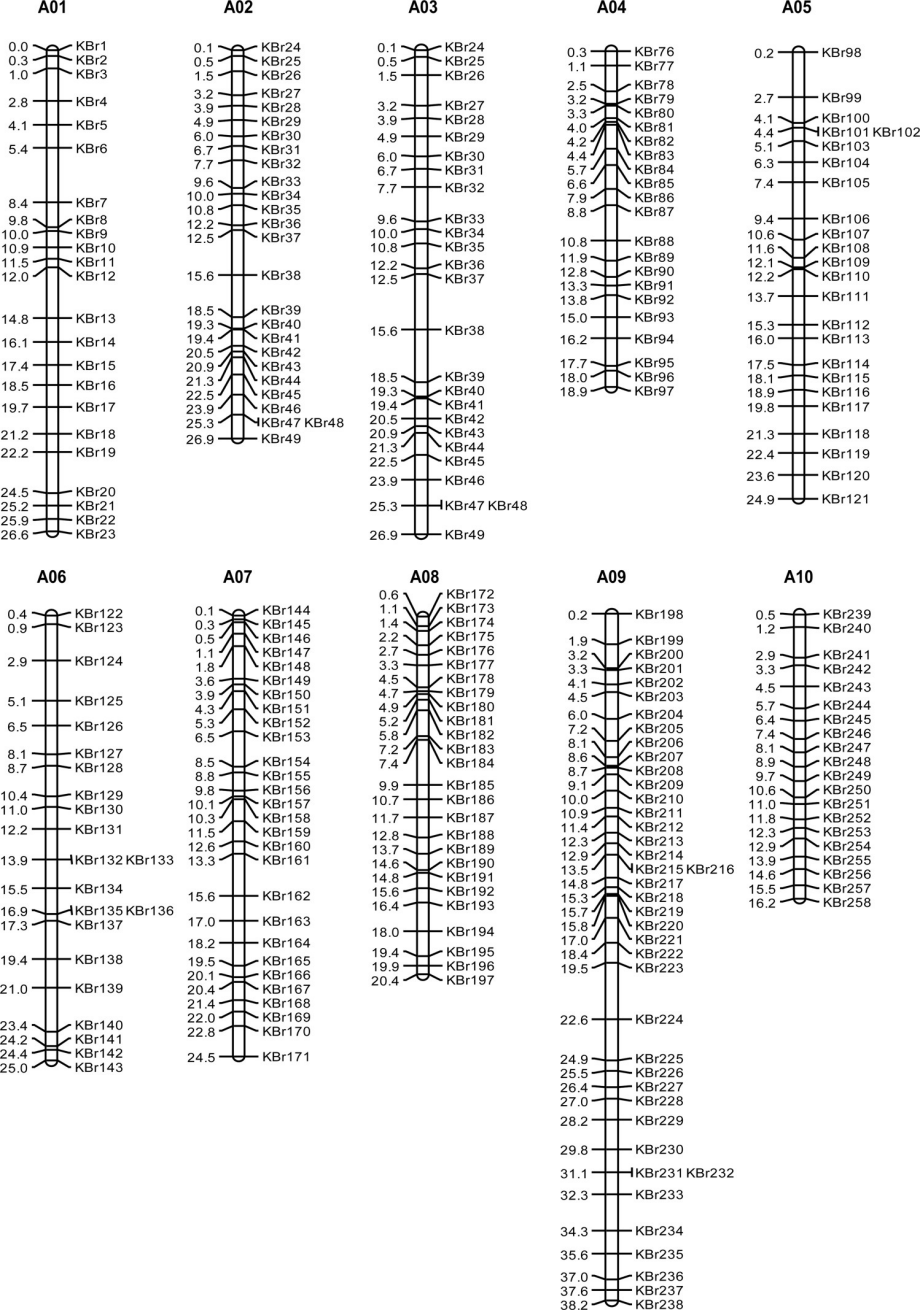
Physical map position of 258 KASP-SNP markers in the Chinese cabbage genome. Marker names are indicated on the right side and physical positions (Mb) on the left side of each chromosome.

Of the 258 polymorphic KASP markers, the number of markers on each chromosome ranged from 20 to 41, with an average of 25.8 markers per chromosome. The marker density along each chromosome ranged from 0.82 to 1.25 markers per Mb, averaging one marker per Mb (Table 3). All of the validated polymorphic KASP markers were distributed evenly along each chromosome, except for a few gaps (Fig 3). Furthermore, 246 (95.35%) KASP makers were identified in genic regions, of which 220 (85.27%) were non-synonymous SNPs, five SNPs gave rise to variants in stop codons, eight were synonymous SNPs, ten were in un-translated regions, and three were in introns. Only 12 (4.65%) markers were in intergenic regions (S7 Table).

**Fig 4:**
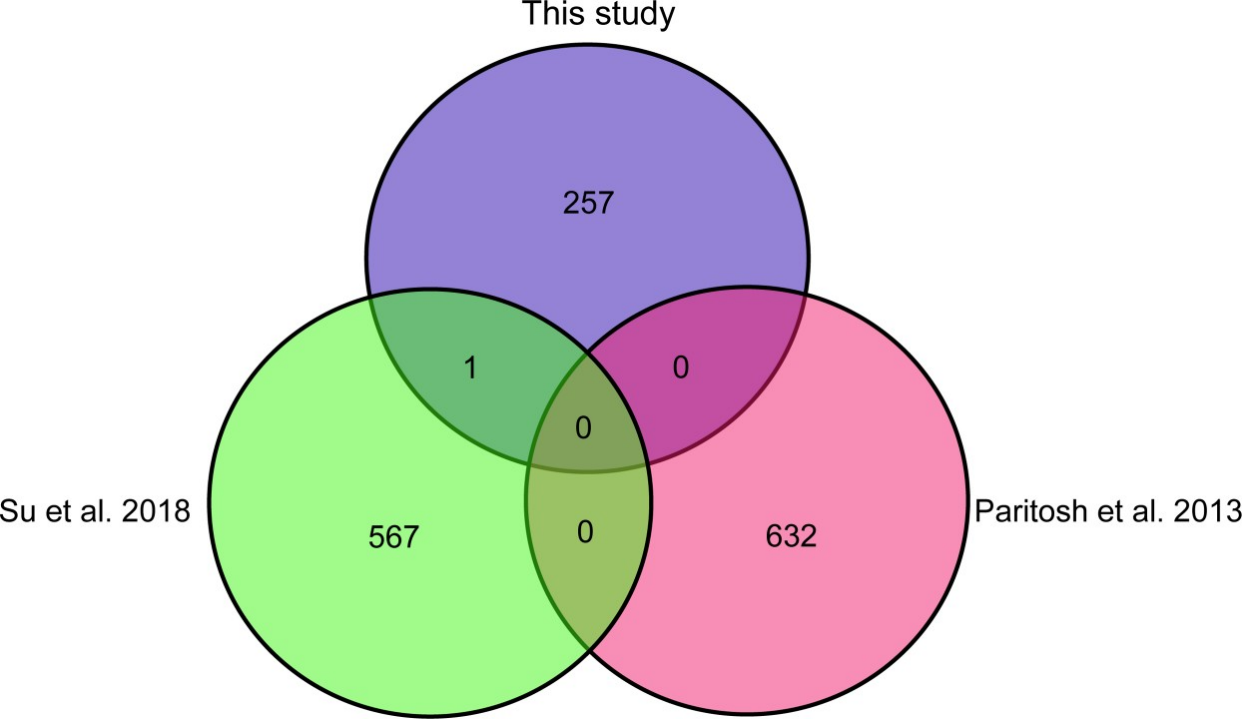
Comparison of KASP-SNP markers with those from previous studies.

Compared with previous published KASP markers[7, 31], only one marker overlapped with [7] (Fig 4), and no one KASP marker overlapped between our study and [31]. This indicates that the 257 developed KASP markers in this study are novel markers.

### Application of KASP markers at population level

**Fig 5:**
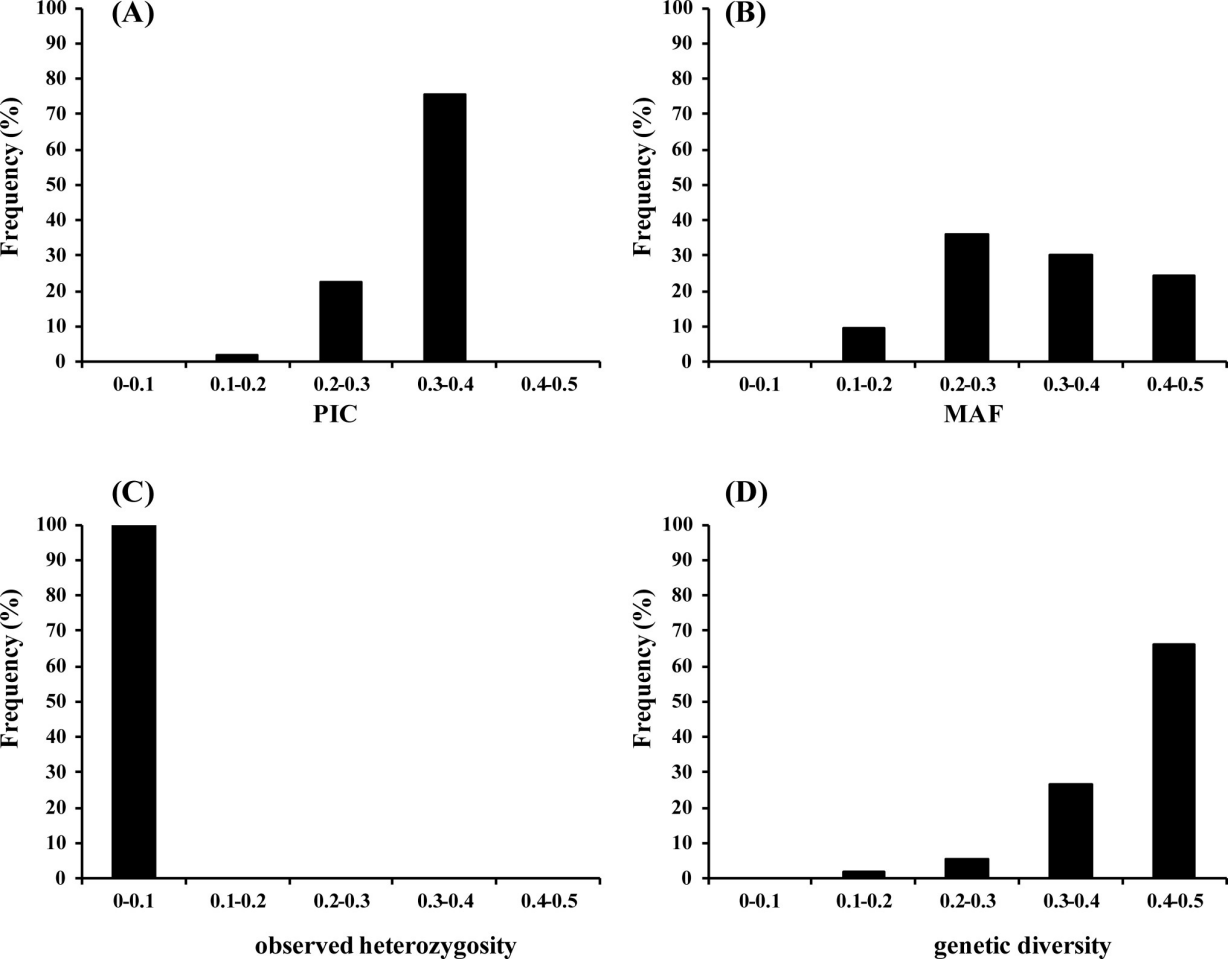
PIC (A), MAF (B), observed heterozygosity (C) and genetic diversity (D) values for the 53 KASP-SNP markers based on data from 34 accessions.

To validate the suitability of the KASP markers, we selected 53 markers, which distributed evenly on each chromosome (S8 Table), for KASP assay genotyping with 34 *Brassica rapa* accessions. The results showed that the PIC value ranged from 0.1676 to 0.3750 with an average of 0.3276 (S9 Table). In particular, the percentage of PIC values between 0.3 and 0.4 was 75% (Fig 5A, S9 Table), which suggested that these markers were strongly polymorphic. The minor allele frequency (MAF) ranged from 0.1029 to 0.5000 with an average of 0.3245 (S9 Table), among which 29 (54.7%) showed a MAF ≥ 0.3 (Fig 5B, S9 Table). All the markers showed an observed heterozygosity < 0.1, with an average of 0.0124 (Fig 5C, S9 Table), which is anticipated because the 34 *Brassica rapa* accessions included are DH lines or inbred lines that have been self-pollinated for many generations and can all be expected to be largely homozygous. The genetic diversity within the germplasm collection was also assessed, which ranged from 0.1847 to 0.5, with an average of 0.4178 (Fig 5D, S9 Table).

**Fig 6:**
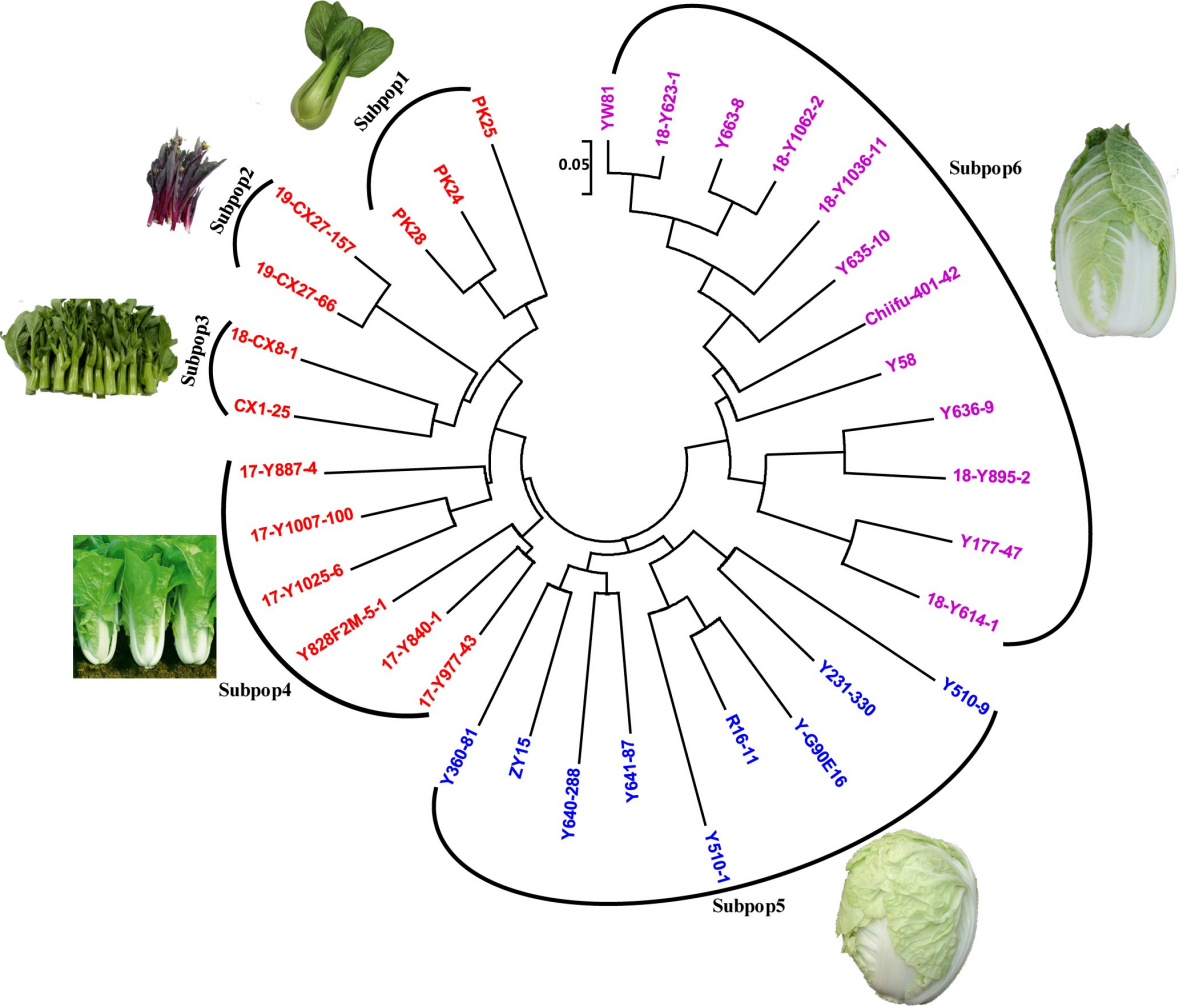
Neighbor-joining tree of 34 *Brassica rapa* accessions calculated from 53 KASP markers. All 34 accessions were further divided into 6 subpopulations. Cluster I, non-heading *Brassica rapa*; Cluster II, over-lapped head type Chinese cabbage; Cluster III, closed head type Chinese cabbage. Subpop1, Pak-choi; Subpop2, Zicaitai; Subpop3, flowering Chinese cabbage; Subpop4, non-heading or half opened head type Chinese cabbage; Subpop5, over-lapped head type Chinese cabbage; Subpop6, closed head type Chinese cabbage.

Based on the genotypes of 53 KASP-SNP markers in 34 *Brassica rapa* accessions (S10 Table), a hierarchical dendrogram was constructed using the neighbor-joining algorithm. All the 34 genotypes were classified into three main clusters based on heading types (Fig 6). Cluster I was further classified into four subgroups: Pak-choi (3 lines), Zicaitai (2 lines), flowering Chinese cabbage (2 lines), half opened head type Chinese cabbage (6 lines) (Fig 6). Cluster II included 9 Chinese cabbage accessions with over-lapped head type (Fig 6). Cluster III comprised 12 Chinese cabbage accessions with closed head type (Fig 6).

## Discussion

### Large-scale SNP loci identification in Chinese cabbage

SNP markers have gained popularity due to their robustness, suitability of automation, and abundance in genomes. Since the genome sequencing data of *Brassica rapa* were published, several genome-wide molecular marker studies have been published. In 2010, 21,311 SNPs were discovered by Sanger sequencing of 1,398 sequence-tagged sites in 8 *Brassica rapa* genotypes with a density of 15.3 SNPs/kb [34]. Based on transcriptome sequencing, Paritosh et al. identified more than 0.2 million SNPs between Chiifu and three oilseed type subspecies [31]; Devisetty et al. detected 330,995 SNPs between R500 (Yellow Sarson) and IMB211 (a rapid cycling variety) with an average frequency of one SNP in every 200 bases [35]; Kim et al. identified 380,198 SNPs among 20 Chinese cabbage accessions [36]. While though genome re-sequencing technology, a total of 1,228,979 SNPs were identified among the 10 non-heading Chinese cabbage accessions with a density of 4.33 SNPs/kb [2], and Su et al. detected a total of 709,037 SNPs among 10 *Brassica rapa* accessions with a density of 2488 SNPs/Mb [7].

In this study, using a whole genome re-sequencing strategy, we detected 827,720 SNPs between Chiifu and four Chinese cabbage DH lines (Y177-47, Y635-10, Y510-1 and Y510-9) with a density of 3,217 SNPs/Mb. The number of SNP loci detected in our study is considerably larger than that detected by Sanger sequencing and transcriptome sequencing [31, 34–36], and is also larger than the number of SNPs in [7]. While the SNP loci are considerably less than those in [2]. This might be attributed to almost 42.53% of the 1,228,979 SNPs being heterozygous loci, while all the SNPs detected in this study are homozygous loci, the heterozygous SNPs have already been filtered out. In terms of SNP frequency, a density of 3,217 SNPs/Mb is within the ranges previously reported for *Brassica rapa* through genome re-sequencing strategy (2,488 SNPs/Mb in [7], and 4,330 SNPs/Mb in [2]), while it is clearly lower than that detected by transcriptome sequencing [31] and Sanger sequencing [30]. The large discrepancy in SNP density appears to be caused by different SNP calling strategies. Compared with the PCR amplicon Sanger sequencing and transcriptome sequencing, *de novo* genomic re-sequencing is more direct and reliable for genomic variation investigations.

A subset of *in silico* SNPs (142 loci) was selected for validation using the Sanger sequencing method, which yielded a 100% SNP validation rate. This result means that almost all of the SNPs found in this study are authentic nucleotide variations in Chinese cabbage. The SNP validation rate is considerably higher than those reported in past studies [16, 17]. Therefore, the large set of putative SNPs mined in this study provides more useful and informative genetic marker resources for Chinese cabbage, and these SNPs can readily be utilized for various maker-based applications in genetics, genomics, and breeding studies in *Brassica rapa*.

### KASP markers are cost-effective and powerful

Numerous methods have been developed for SNP genotyping, including allele-specific PCR (AS-PCR), cleaved amplified polymorphic sequences (CAPS) and high-resolution melting (HRM) curve assays. All these methods require gel electrophoresis or high-resolution melting to separate products, which are low throughput, high cost, and labor intensiveness [8]. A number of high-throughput SNP genotyping platforms have also been developed, such as BeadXpress, GoldenGate and Infinium from Illumina [37], TaqMan assay [38] and genotyping by sequencing (GBS) [39]. The methods developed for high throughput, with cost being a secondary issue, could not be carried out by all regular laboratories. KASP technology has the advantages of high reliability and cost-effectiveness, and it has scalability that makes it suitable for a wide range of experimental designs with greatly varying target loci and sample numbers [31, 37, 40]. KASP is easier to use: either LGC Genomics can provide a full KASP genotyping service, or the KASP reagents can be ordered from them for performing assays in a basic molecular laboratory [23].

In this study, we established a KASP-SNPs resource which comprised 387,354 SNPs with at least 30 bp to the next most adjacent SNPs on either side. From this resource, we selected 268 SNP loci, designed primers and carried out KASP assays using Roche LightCycler 480-II instrument. In total, 258 (96.27%) were successfully converted into KASP markers, the marker conversion rate was considerably higher than SSR markers (28.44%) [41], CAPS markers (70.06%) [42] and HRM markers (65.45%) [43]. Furthermore, the KASP marker conversion rate is also higher than that reported in wheat (78.00%) [44] and chickpea (80.65%) [22] using the same technology. The possible reasons for this could be (a) a reference genome with high quality is available in *Brassica rapa*, (b) the SNP loci identified in this study are mostly authentic, and (c) stringent criteria were used for selecting SNP loci.

Co-dominant SNP assays are preferred markers compared with dominant SNP assays due to the ability to differentiate heterozygotes from the homozygous genotype in segregant populations, such as F_2_ and backcross populations [45]. Of the 258 KASP markers developed in this study, 221 (85.66%) were co-dominant markers, which will be valuable for genetic and genomic studies in Chinese cabbage. The SNP markers should theoretically all be co-dominant, while 37 (14.34%) KASP markers in our study showed features of dominant markers. The potential reasons for this phenomenon might be caused by the weak amplification ability of one allele-specific primer. Because one allele-specific primer amplified weak production, while the other one produced strong signal, so the heterozygous genotype was much closer to the stronger one. During our daily experiment, we have converted one dominant KAPS marker into co-dominant marker through optimization of primer which had weak amplification ability (S1 Fig).

Compared to non-coding genomic markers, SNPs developed from functional genes are potentially more closely related to phenotypic variations [34, 46]. In our study, 220 (85.27%) KASP markers were located in exons and altered the amino acid sequence encoded, and so could be used as functional markers.

Of all 258 KASP markers developed in this study, 257 are novel SNP markers, which extended the KASP marker resource in *Brassica rapa*, and could be used for high-density genetic linkage map construction, gene/QTL mapping, cultivar identification, and so forth.

### Genetic diversity in the 34 *Brassica rapa* accessions

A total of 53 KASP markers were selected for KASP assay genotyping with 34 *Brassica rapa* accessions. The 53 KASP markers produced 106 allelic variations with 2 alleles per SNP. The PIC values ranged from 0.1676 to 0.3750 with an average of 0.3276, which is much higher than recently developed KASP markers for chickpea (0.12) and Grasspea (0.2457) [18, 22], and comparable to that for *Brassica rapa* (0.35) [7]. The average of MAF and genetic diversity values were 0.3245 and 0.4178, which also suggested a high discriminatory ability for these SNPs. A neighbor-joining tree based on CSChord (1967) genetic distance matrix separated 34 *Brassica rapa* accessions into three distinct clusters, which matched with the *Brassica rapa* heading types. Materials in cluster I were all non-heading type. Three Pak-choi, two Zicaitai and two flowering Chinese cabbage were classified into three subgroups based on variety distinctness and were much closer to each other than to Chinese cabbage, which is consistent to previous studies [6, 7]. It is interesting that six accessions consumed as seeding vegetables, with high growth rate, non-heading or half opened head type, belonged to Chinese cabbage, were much closer to non-heading Chinese cabbage group. It can be anticipated because heading or non-heading is the most distinctive characteristic between Chinese cabbage and non-heading Chinese cabbage, which also confirms the evolution theory of Chinese cabbage [3, 4]. Nine over-lapped head type Chinese cabbage and twelve closed head type Chinese cabbage were grouped into two different clusters. Su et al. classified 99 Chinese cabbage lines into three clusters based on cultivation season: spring, summer and autumn-ecotype [7]. Usually, summer-ecotype are over-lapped head type, spring and autumn-ecotype in that study are closed head type (personal communication), which suggested an consistence between these two studies.

## Conclusion

In summary, We re-sequenced 4 Chinese cabbage lines and obtained more than 38.5 billion high quality sequenced bases. Based on genome-wide SNP detection and validation through Sanger sequencing, we identified 827,720 SNP loci with a high accuracy rate. A total of 387,354 SNPs with at least 30 bp to the next most adjacent SNPs on either side were selected as a KASP-SNPs resource. Among which, 258 SNP loci were successfully transformed into KASP markers, the marker conversion rate was high to 96.27%. Additionally, 221 (85.66%) were co-dominant markers, 220 (85.27%) were non-synonymous SNPs, and 257 (99.6%) were newly developed markers. Thirty four *B*.*rapa* accessions genotyped with 53 KASP markers can be separated into three distinct clusters, which matched with the *Brassica rapa* heading types. Furthermore, three Pak-choi, two Zicaitai and two flowering Chinese cabbage were divided into three subgroups according to varieties, which demonstrated KASP markers mined here are not only useful in Chinese cabbage, but also for other *Brassica rapa* crops. The large set of SNPs discovered in this study and newly developed KASP markers will be useful for genetic improvement of Chinese cabbage through breeding.

## Supporting information

S1 Appendix Flanking sequence of 387354 KASP-SNPs. (FASTA)

S1 Fig A KASP marker was converted from dominant to co-dominant. Primer sequences (A) and KASP genotyping (C) of a dominant KASP marker, optimized primer sequences (B) and KASP genotyping (D) with co-dominant feature.
(TIF)

S1 Table 34 *Brassica rapa* accessions used in this study. (XLSX)

S2 Table Number and density of SNPs on each chromosome. (XLSX)

S3 Table Number of SNPs in pairwise comparisons of the four Chinese cabbage DH lines and the reference genome Chiifu-401-42. (XLSX)

S4 Table Sanger sequencing primers for SNP validation. (XLSX)

S5 Table Validation of selected SNPs by comparison between re-sequencing and Sanger sequencing. (XLSX)

S6 Table Primer sequences and genetic information for KASP markers developed in this study. (XLSX)

S7 Table Classification of 258 KASP-SNP markers in genic and intergenic regions. (XLSX)

S8 Table Information of 53 KASP markers selected for diversity analysis. (XLSX)

S9 Table Summary of 53 KASP marker diversity in 34 *Brassica rapa* accessions. (XLSX)

S10 Table Genotypes of 53 KASP-SNP markers in 34 *Brassica rapa* accessions. (XLSX)
