# Supplementary figures and images for: An extended KASP-SNP resource for molecular breeding in Chinese cabbage(Brassica rapa L. ssp. pekinensis)
Source: PLoS One. 2020 Oct 2;15(10):e0240042. doi: 10.1371/journal.pone.0240042 (PMC7531813; doi:10.1371/journal.pone.0240042)

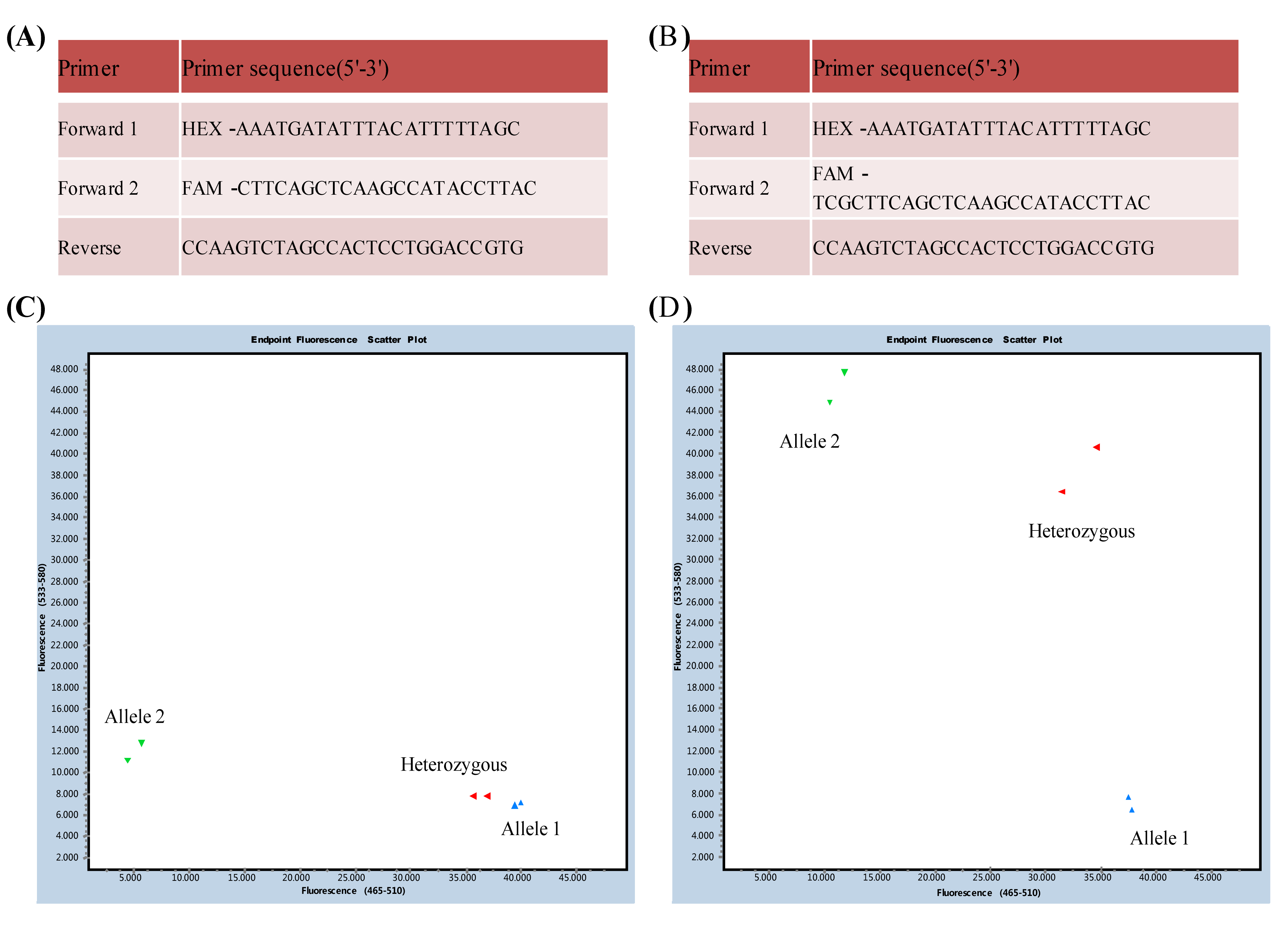

Supplement: S1 Fig — Primer sequences (A) and KASP genotyping (C) of a dominant KASP marker, optimized primer sequences (B) and KASP genotyping (D) with co-dominant feature. (TIF) [file pone.0240042.s002.tif]
